# Anti-VEGF Treatment of Diabetic Macular Edema: Two-Year Visual Outcomes in Routine Clinical Practice

**DOI:** 10.1155/2020/6979758

**Published:** 2020-03-16

**Authors:** Mojca Urbančič, Pia Klobučar, Matej Zupan, Katja Urbančič, Alenka Lavrič

**Affiliations:** ^1^Eye Hospital, University Medical Centre Ljubljana, Grablovičeva 46, 1000 Ljubljana, Slovenia; ^2^Faculty of Medicine, University of Ljubljana, Vrazov Trg 2, 1000 Ljubljana, Slovenia

## Abstract

**Purpose:**

The purpose of this study was to evaluate 2-year visual outcomes in patients with diabetic macular edema (DME) treated with anti-VEGF agents in a routine clinical setting.

**Methods:**

The medical records of patients treated with ranibizumab or aflibercept due to DME at the Eye Hospital, University Medical Centre Ljubljana, Slovenia, between January 2016 and March 2019 were retrospectively reviewed. After applying inclusion and exclusion criteria, 123 patients (123 eyes) were included in the study.

**Results:**

Baseline visual acuity (VA) was 60.9 ± 15.2 letters (median 63; range 7–85). Baseline central retinal subfield thickness (CRT) was 440.7 ± 132.5 *μ*m (median 430; range 114–1000). No significant change in VA over 2 years was found (mean change +2.1 ± 16.8 letters (median 2; range −53–52)). However, there was a significant change in VA in the subgroup with baseline VA <70 letters (mean change +5.7 ± 17.9 letters (median 5; range −52–52)). VA gains of ≥15 letters were achieved in 25 eyes (20.3%). Changes in CRT were significant over 2 years. Patients received 4.5 ± 2.1 (median 5, range 1–9) and 2.6 ± 2.3 (median 2, range 0–8) injections in the first and second years, respectively.

**Conclusions:**

The two-year visual outcomes in this retrospective analysis appear to be comparable to previously reported outcomes in routine clinical practice. Our analysis provides some information about the effectiveness of anti-VEGF treatment in routine clinical practice in Slovenia. More intensive treatment should be implemented in the management of patients in order to achieve better visual outcomes.

## 1. Introduction

Diabetic macular edema (DME) may affect up to 7% of patients with diabetes. This vision-threatening complication of diabetes can have a significant impact on patient quality of life. The risk factors for DME development are largely similar to those of diabetic retinopathy (DR) [1, 2].

Vascular endothelial growth factor (VEGF) has a crucial role in the complex pathogenesis of DME [3–5]. One of the most obvious effects of VEGF activity is the blood-retinal barrier breakdown [6]. Agents that block VEGF action restore the integrity of the blood-retinal barrier, resolve macular edema, and improve vision in most patients with DME [7]. Several randomized clinical trials (RCTs) have demonstrated the efficacy and safety of anti-VEGF agents in the treatment of DME, with greater improvements in visual acuity (VA) achieved by anti-VEGF treatment compared to laser therapy [8–12]. The introduction of anti-VEGF agents into clinical practice has considerably changed the management of patients with DME. Currently, intravitreal anti-VEGF agents are the preferred first-line treatment for DME [13]. Corticosteroids can also be used in the management of DME, mostly as a second-line treatment option [14–19].

RCTs, the gold standard for evaluating treatment outcomes, are research tools with strong internal validity but low generalizability to real-life conditions [20]. It is difficult to implement intensive RCT treatment protocols in routine clinical practice, where patient selection is not as rigorous and resources differ from those in RCTs [21]. In contrast to RCTs, real-life studies reflect the management of patients in routine clinical practice and provide insight into the real-life effectiveness of treatment [20]. Most real-life studies evaluating the effectiveness of anti-VEGF treatment in DME have demonstrated lower VA gains in comparison with RCTs [22–28].

Anti-VEGF treatment of patients with DME in Slovenia started in 2011. There is a constant overload of patients needing anti-VEGF treatment. The difficulties in managing an increasing number of patients may have an impact on treatment results. The purpose of this study was to evaluate 2-year visual outcomes in patients with DME treated with anti-VEGF agents in a routine clinical setting at the Eye Hospital, University Medical Centre Ljubljana, Slovenia.

## 2. Methods

The medical records of all patients treated with an anti-VEGF agent (ranibizumab or aflibercept) for DME at the Eye Hospital, University Medical Centre Ljubljana between January 2016 and March 2019 were retrospectively reviewed. Data collected were age, history of previous treatment for DME, best-corrected VA at baseline and one year and two years of follow-up, central retinal subfield thickness (CRT) at baseline and one year and two years of follow-up, morphological type of the edema on optical coherence tomography imaging (OCT), presence of vitreomacular traction, stage of DR, prior laser treatment (laser treatment for macular edema and/or panretinal photocoagulation), number of visits and number of anti-VEGF injections in the first and second year, and adverse events.

The inclusion criteria were patients older than 18 years, a diagnosis of DME, availability of complete ophthalmological medical records, and a follow-up period of at least 2 years. The exclusion criteria were incomplete ophthalmological data, significant vitreomacular traction, other ocular conditions that could affect VA, laser treatment or treatment with steroids less than 6 months prior to anti-VEGF treatment and/or during the follow-up period, cataract surgery during the follow-up period and vitrectomy. If patients received treatment in both eyes, only one eye, randomly chosen, was included in the present analysis. Randomization was digitalized. The researchers who collected the data were not involved in the management of the patients.

Patients were managed according to routine clinical practice. A complete ophthalmological examination (VA testing, slit lamp and dilated fundus examinations, intraocular pressure measurement), OCT, fundus photography, and fluorescein angiography were performed at the first visit to evaluate DME and the stage of DR before any treatment decision. All patients signed informed consent to the treatment and to the use of their anonymized data for the purposes of clinical audit and research.

A pro re nata (PRN) treatment regimen was supposed to be implemented for anti-VEGF treatment after three to five monthly injections (depending on the drug that was used) as a loading phase. A complete ophthalmological examination, fundus photography, and OCT were performed at every follow-up visit. VA testing was performed using an ETDRS chart (4 meter 2000 series revised ETDRS chart (Precision Vision®, La Salle, USA)), and the best-corrected VA was recorded as the number of ETDRS letters. CRT was measured automatically by a SD-OCT machine 3D-OCT 1000 (Topcon Corp.®, Tokyo, Japan). Nurses trained in ETDRS visual acuity testing tested VA according to the international standards for ETDRS visual acuity testing. An OCT image of the macula was taken by a trained photographer. Nurses and photographers changed according to their work schedule, so each patient at each visit was randomly assigned to a certain nurse or photographer. Each patient was managed by the same physician at every visit.

The baseline characteristics of the patients were noted. The mean VA and mean CRT at 1 year and 2 years were compared to those of the baseline. The mean change in VA and mean change in CRT at 1 year and 2 years were calculated. The proportions of eyes with a VA gain or loss of ≥10 letters and ≥15 letters were also calculated. Eyes with a VA ≥70 letters and eyes with a complete resolution of edema were noted. The number of injections and the number of visits was noted as well.

Eyes were divided into two subgroups according to baseline VA (group 1 with baseline VA <70 letters, group 2 with baseline VA ≥70 letters). The mean VA and mean CRT at 1 year and 2 years were compared to those of the baseline for each group. The mean changes in VA, mean changes in CRT, and the proportions of eyes with a VA gain or loss of ≥10 letters and ≥15 letters were calculated for each group at 1 year and 2 years.

### 2.1. Statistical Analysis

Descriptive statistics included the mean with standard deviation and median with range (minimum and maximum value) for numerical variables. Since the data did not meet the normality assumption, nonparametric tests were used to assess the differences: the Friedman test and Wilcoxon signed-rank test were used for evaluating changes in the variables from baseline to 1 year and 2 years. The Mann–Whitney *U* test was used to test the differences in the data between the subgroups of eyes. Additionally, a repeated measures test was used to test the differences in VA and CRT over time and between the subgroups. The McNemar test was used to compare the proportions of eyes gaining or losing ≥10 letters and ≥15 letters. A *p* value less than 0.05 was considered statistically significant. Statistical analyses were performed using SPSS version 21 (SPSS IBM, New York, USA).

The study adhered to the tenets of the Declaration of Helsinki and was approved by the Slovenian National Medical Ethics Committee (National Medical Ethics Committee number 0120-604-2018).

## 3. Results

The medical records of all 228 patients (303 eyes) receiving anti-VEGF treatment for DME between January 2016 and March 2019 were reviewed. After applying the inclusion and exclusion criteria, 123 patients (123 eyes) were included in the study. There were 32 eyes treated with ranibizumab, 51 eyes treated with aflibercept, and 40 eyes that received both drugs during the 2-year period (at some time point, one drug was changed for the other).

### 3.1. Baseline Characteristics

The mean age of the patients was 67.5 ± 8.8 years, 80 were men (65%), and 43 were women (35%). OCT evaluation of the cases of DME showed diffuse edema in two eyes (1.6%), cystoid edema in 56 eyes (45.5%), and edema with a serous detachment in 65 eyes (52.8%). Mild to moderate nonproliferative DR was present in 21 eyes (17.1%), severe nonproliferative DR in 69 eyes (56.1%), and proliferative DR in 33 eyes (26.8%). Prior laser treatment of DME (laser photocoagulation or subthreshold micropulse laser treatment) was performed in 78 eyes (63.4%). Panretinal photocoagulation or some peripheral laser photocoagulation treatment was performed in 46 eyes (37.4%) before the start of DME treatment with an anti-VEGF agent. The baseline VA was 60.9 ± 15.2 letters (median 63; range 7–85). The baseline CRT was 440.7 ± 132.5 *μ*m (median 430; range 114–1000).

### 3.2. VA

**Figure 1:**
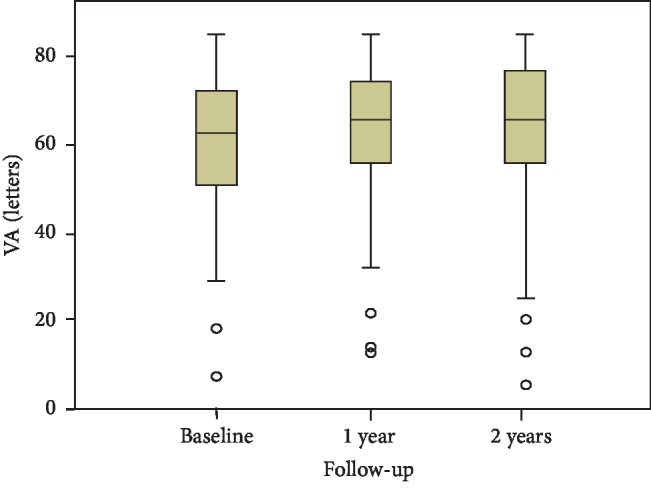
The VA at baseline, 1 year, and 2 years (all 123 eyes)—the changes were not significant (Friedman test: *p*=0.471).

The VA at baseline, 1 year, and 2 years and the VA changes between baseline and 1 year and between baseline and 2 years are presented in Table 1. There was no statistically significant improvement in VA at 1 year or 2 years (Friedman test; *p*=0.471, Figure 1). The proportions of eyes with a VA gain of ≥10 letters and a VA gain of ≥15 letters and the proportions of eyes with a VA loss of ≥10 letters and a VA loss of ≥15 letters are presented in Table 2. There were 22 eyes (17.8%) with a baseline VA ≤45 letters and 46 eyes (37.4%) with a VA ≥70 letters at baseline. The proportion of eyes with a VA ≥70 letters increased to 53 (43.1%) and 56 (45.5%) at 1 year and 2 years, respectively.

### 3.3. CRT

**Figure 2:**
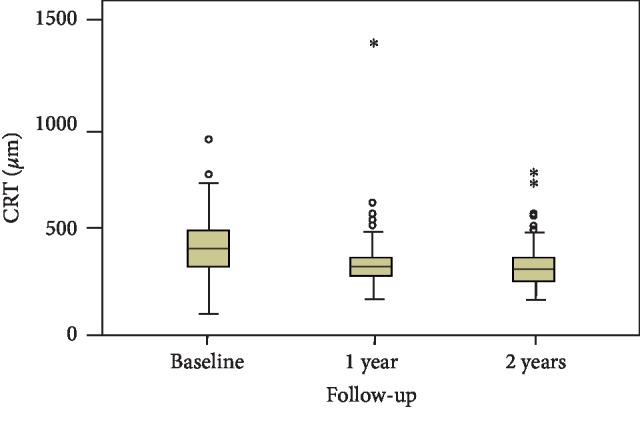
The CRT at baseline, 1 year, and 2 years (all 123 eyes)—the changes were significant (Friedman test: *p* < 0.0001), significantly different changes between all observed time points (Wilcoxon signed-rank test: *p* < 0.0001).

The CRT at baseline, 1 year, and 2 years and the CRT changes between baseline and 1 year and between baseline and 2 years are presented in Table 3. The change in CRT was statistically significant (Friedman test; *p* < 0.0001, Figure 2), and the Wilcoxon signed-rank test revealed significantly different changes between all observed time points (*p* < 0.0001). There were 70 eyes (56.9%) and 81 eyes (65.8%) with a CRT reduction of ≥10% at 1 year and 2 years, respectively. A CRT less than 250 *μ*m was documented in 11 eyes (8.9%) at 1 year and in 18 eyes (14.6%) at 2 years.

### 3.4. Number of Visits and Injections

Patients had 6.7 ± 1.4 (median 7, range 4–11) visits in the first year and 6.5 ± 1.2 (median 6, range 4–10) in the second year (Wilcoxon signed-rank test: *p*=0.007). The patients received 4.5 ± 2.1 injections (median 5, range 1–9) in the first year and 2.6 ± 2.3 (median 2, range 0–8) in the second year (Wilcoxon signed-rank test: *p* < 0.0001).

### 3.5. Analysis of Subgroups according to Baseline VA

**Figure 3:**
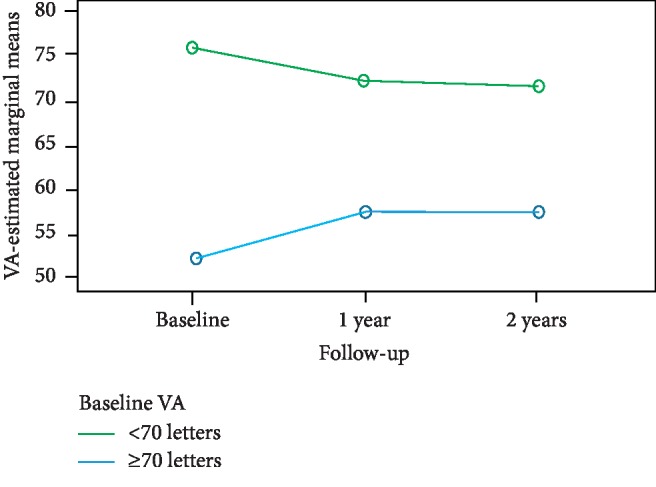
VA change over time for the subgroups divided according to baseline VA. The between-subgroups test was significant (*p* < 0.0001); the lines for the two subgroups are rather far apart in the graph. The within-subject test indicates that there was no overall significant time effect (*p*=0.579). However, there was an interaction between the subgroups and time (*p*=0.002): the line representing the subgroup with baseline VA <70 letters increases over time. In contrast, the line representing the subgroup with baseline VA ≥70 letters slightly decreases over time.

**Figure 4:**
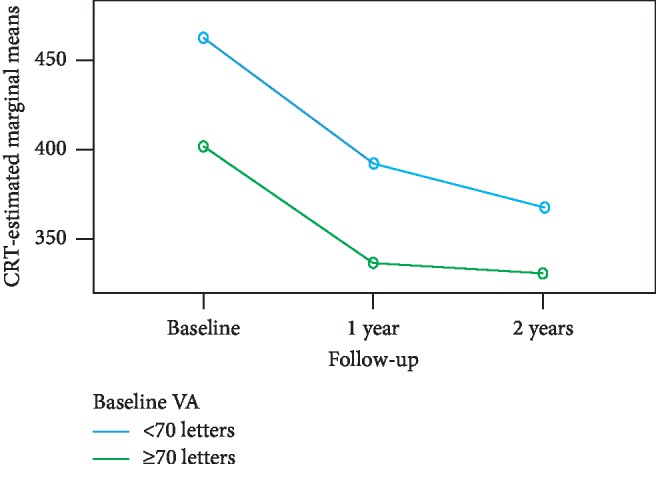
CRT change over time for the subgroups divided according to baseline VA. The between-subgroups test was significant (*p*=0.004); the lines for the two subgroups are rather far apart. The within-subject test indicated a significant time effect (*p* < 0.001): CRT decreases over time. The interaction between the groups and time was not significant (*p*=0.272): CRT similarly decreases over time in both subgroups.

Analysis of subgroups according to baseline VA showed no statistically significant changes in VA during the follow-up period in patients with baseline VA ≥70 letters (Friedman test: *p*=0.195, Table 1). In contrast, there were statistically significant changes in VA during the follow-up period in the subgroup with baseline VA <70 letters (Friedman test: *p*=0.017, Table 1): the changes were significant between baseline VA and VA at 1 year (Wilcoxon signed-rank test: *p*=0.015) and between baseline VA and VA at 2 years (Wilcoxon signed-rank test: *p*=0.003).  Figure 3 shows the VA change over time for the subgroups divided according to baseline VA. The proportions of eyes with a VA gain of ≥10 letters and a VA gain of ≥15 letters and the proportions of eyes with a VA loss of ≥10 letters and a VA loss of ≥15 letters for both subgroups are presented in Table 2. There were statistically significant changes in CRT from baseline to 1 year and 2 years, respectively, in both subgroups according to baseline VA (Friedman test: *p* < 0.0001; Wilcoxon signed-rank test: *p* < 0.0001, Table 3).  Figure 4 shows the CRT change over time for the subgroups divided according to baseline VA.

Eyes with a baseline VA <70 letters received 4.2 ± 1.9 injections (median 4, range 1–8) in the first year and 2.3 ± 2.3 (median 2, range 0–8) in the second year. Eyes with a baseline VA ≥70 letters received 5.1 ± 2.3 injections (median 5, range 1–9) in the first year and 3.1 ± 2.3 (median 3, range 0–8) in the second year. Eyes with a lower baseline VA received significantly fewer injections in the first year (Mann–Whitney *U* test: *p*=0.004). There were no statistically significant differences in the number of injections between the groups in the second year (Mann–Whitney *U* test: *p*=0.26).

### 3.6. Serious Adverse Events

No serious adverse events were noted during the follow-up period.

## 4. Discussion

Our retrospective analysis showed no significant change in VA over 2 years (mean change +2.1 ± 16.8 letters; median 2; range −53–52). However, there was a significant change in VA in the subgroup with a baseline VA <70 letters (mean change +5.7 ± 17.9 letters; median 5; range −52–52). VA gains of ≥15 letters were achieved in 25 eyes (20.3%). Changes in CRT were significant over 2 years in all eyes and in both subgroups divided according to baseline VA. These results were achieved with 4.5 ± 2.1 (median 5, range 1–9) and 2.6 ± 2.3 (median 2, range 0–8) injections in the first and second years, respectively.

The mean VA gain in our routine clinical practice was lower than the VA gains achieved in RCTs. Trials such as the RISE and RIDE, DRCR.net Protocol I, RESTORE, RESOLVE, VIVID, and VISTA, and Protocol *T* trials [8, 11, 29–32] demonstrated VA gains of +6.1 to +13.3 letters over 1 year. At 2 years, similar VA gains (+6.0 to +12.8 letters) were observed in the RISE and RIDE, DRCR.net Protocol I, RESTORE Extension Study, VIVID and VISTA, and Protocol *T* trials [10, 11, 29, 33, 34]. There could be several reasons for not achieving similarly high VA gains in our routine clinical practice. First, a large number of eyes had already undergone previous laser treatment for DME, which suggests the possibility of chronic DME, where anti-VEGF agents might not be very effective. Second, some eyes had very low baseline VA, suggesting possible morphological changes associated with permanent VA loss. On the other hand, 37.4% of eyes had baseline VAs better than 70 letters, which presumably had an impact on VA gain due to the ceiling effect. If these eyes were excluded from the analysis, the VA gain became significant, although still lower than in RCTs. Finally, and probably of crucial importance, our patients had fewer visits and received fewer injections during the observed period in comparison to the patients in the RCTs.

More than half of the eyes (63.4%) included in this retrospective review were not treatment naïve. Data regarding the duration of DME were incomplete and were not included in the present analysis. We do not know how many of these eyes had chronic edema, but we speculate that a significant number of treated eyes were poor responders to anti-VEGF treatment. In persistent DME not responding to anti-VEGF treatment, it is reasonable to switch to corticosteroids [13–19, 35, 36]. However, information about factors influencing the physician's decision to continue with anti-VEGF treatment in an individual case could not be found in our retrospective data. We can assume that the treatment response was good, but that the overall result of treatment was not optimal due to undertreatment.

Twenty-two eyes (17.8%) had baseline VAs ≤45 letters (equivalent to ≤20/125) in our retrospective analysis. Channa and coworkers analyzed factors affecting visual outcomes in patients with DME treated with ranibizumab and concluded that poor baseline VA (≤20/125) predicts poor visual outcome (≤20/100) after 2 years of treatment with ranibizumab and/or laser [37]. Similarly, Sophie and coworkers found that a low baseline VA was associated with poor visual outcome [38]. Low VA is often associated with chronic edema and permanent damage of the retina [39]. However, our retrospective analysis did not include analysis of possible correlations between OCT structural changes and VA. Therefore, the influence of eyes with low baseline VA on mean VA gain in this study remains unclear.

Eyes with good baseline VA have lower VA gain due to ceiling effect, which is evident from our results. Of the eyes with a VA <70 letters, 32.5% had a VA gain of ≥15 letters in our study, which might be comparable to RCTs such as the RISE and RIDE trials and the VIVID and VISTA trials [11, 29], despite the significantly higher number of injections administered in these trials. Patients eligible for the RISE and RIDE trials had VAs between 20/40–20/320 (20/40 ≈ 70 letters), and the proportions of patients gaining ≥15 letters at 2 years were 33.6–45.7% [29]. The VIVID and VISTA trials had the same VA enrolment criteria, and 31.1–38.3% of patients gained ≥15 letters at 100 weeks [11]. Our patients received 7.1 injections in 2 years in contrast to the 24 injections administered in the RISE and RIDE trials [29] or the 13.5–22.6 injections in the VIVID and VISTA trials [11]. Patients enrolled in the RESTORE Study had a baseline VA 79–39 letters, received on average 7 injections over a 1-year period and gained +6.8 letters in the ranibizumab monotherapy subgroup [8]. Notably, subgroup analysis in the same trial showed a VA gain of only +2.1 letters in patients with a baseline VA greater than 73 letters [8], which clearly indicates the importance of considering baseline VA when interpreting VA outcomes. In contrast to the RESTORE Study, where 19.8% of patients had a baseline VA >73 letters, 37.4% of patients had a baseline VA ≥70 letters in our retrospective analysis. The effect of baseline VA on VA gain was clearly demonstrated by Dugel and coworkers, who conducted a cross-trial comparison on data from nine clinical trials and found that mean VA gain negatively correlated with baseline VA [40].

Kodjikian with coworkers analyzed 32 real-life studies evaluating the efficacy of anti-VEGF agents in the management of DME. The patients had a mean baseline VA of 57.3 letters (range 38–72 letters). The mean follow-up was 15.6 months (6–48 months). During follow-up, a mean VA gain of +4.7 letters (−5–+8.5 letters) was observed for a mean of 5.8 injections (1.3–17). The mean final VA was 62 letters (42–77.5 letters) [41]. These summarized results are in concordance with our results when considering only eyes with a baseline VA <70 letters, where mean VA gains of +5.3 letters and +5.7 letters at 1 year and 2 years, respectively, were observed. The mean number of injections in our analysis also tended to be similar to these summarized results. Similarly, a large prospective noninterventional OCEAN Study, which evaluated the use of ranibizumab in a routine clinical setting, demonstrated mean VA gains of +4 letters and +5.2 letters at 1 year and 2 years, respectively. Although the mean VA gains were lower in our analysis, similar proportions of patients gained ≥15 letters (23.5% in the OCEAN Study vs. 20.3% in our analysis) or lost ≥15 letters (7% vs. 9.7%) at 2 years [22].

Although fluctuations in VA and CRT were noticed during the follow-up period in our retrospective review, only the data at three time points (baseline, at 1 year and at 2 years) were included in the final analysis. In a retrospective study performed by Wecker and coworkers, the mean maximum VA gain during the first year was +6.2 letters. Maximum VA gain, however, occurred at different time points for each patient. As a result, the mean VA change for any given time point was less pronounced. By the end of the first year, the mean VA was -1.3 letters [42]. Our results might have been more favorable if the mean maximum VA gain had been considered.

Based on the comparison between RCTs and observational real-life studies evaluating anti-VEGF treatment, it appears that visual outcomes are strongly correlated with the number of injections. Patients treated with anti-VEGF injections in a routine clinical practice receive a substantially lower number of injections in comparison to patients included in RCTs [41]. The mean number of injections in a 2-year period in our analysis was 7.1 ± 3.6 injections (median 7, range 1–17), which is 2-3 times less than in RCTs [10, 11, 29, 34]. Furthermore, the mean number of visits in a 2-year period in our routine clinical setting was 13.4 ± 2.4 (median 13, range 8–20), which is not in accordance with Slovenian and European guidelines for the management of DME [13, 43]. Although the PRN regimen was the recommended protocol, patients were not followed on a monthly basis and consequently could not receive monthly injections if needed. Since only patients with 2 years of follow-up were included in our retrospective analysis, there were no patients lost to follow-up that could have influenced the final results. Some of the reasons for the low number of visits could be patient comorbidities or transportation problems. However, the most obvious reasons are the limited capabilities of the hospital to provide timely treatment for all patients.

Our analysis has some limitations, such as its retrospective nature, the inclusion of eyes with very low or very good baseline VA and the involvement of many physicians with different clinical experiences and sometimes variable retreatment criteria. However, the study provides information about the real-life effectiveness of anti-VEGF treatment, represents the first analysis of the effectiveness of anti-VEGF treatment in Slovenia, and can serve to improve the quality of management of our patients.

## 5. Conclusions

The two-year visual outcomes in this retrospective analysis appear to be less favorable compared to previously reported outcomes when considering only VA gain, although comparable proportions of eyes gaining ≥15 letters have been observed. A large proportion of our patients had a baseline VA ≥70 letters, which must be taken into account when interpreting the results. When only eyes with a VA <70 letters are considered, the results seem more comparable to the outcomes from other studies. Our analysis provides some information about the effectiveness of anti-VEGF treatment in routine clinical settings in Slovenia. Most importantly, this indicates that more intensive treatment should be implemented in the management of patients to achieve better visual outcomes.

## Figures and Tables

**Table 1:** The VA at baseline, 1 year, and 2 years.

VA, mean ± SD (median; range) (ETDRS letters)						
	Baseline	1 year	Change from baseline at 1 year	2 years	Change from baseline at 2 years	*p* value
All eyes (*n* = 123)	60.9 ± 15.2 (63; 7–85)	62.9 ± 15.3 (66; 13–85)	+2.2 ± 14.5 (1; −41–48)	62.9 ± 16.9 (65; 4–85)	+2.1 ± 16.8 (2; −53–52)	0.47
Eyes with baseline VA <70 letters (*n* = 77)	52.1 ± 12.3 (54; 7–69)	57.3 ± 16.0 (59; 13–85)	+5.3 ± 16.7 (4; −41–48)	57.6 ± 17.4 (60; 4–85)	+5.7 ± 17.9 (5; −52–52)	0.017
Eyes with baseline VA ≥70 letters (*n* = 46)	75.6 ± 4.6 (75; 70–85)	72.4 ± 7.6 (72.5; 58–85)	−2.9 ± 7.4 (−2; −21–10)	71.7 ± 11.9 (75; 32–85)	−3.9 ± 12.6 (−2; −53–11)	0.11

Legend: VA = visual acuity.

**Table 2:** The proportions of eyes gaining or losing ≥10 letters and ≥15 letters.

Number of eyes (percentage)												
	VA gain ≥10 letters			VA gain ≥15 letters			VA loss ≥10 letters			VA loss ≥15 letters		
	1 year	2 years	*p* value	1 year	2 years	*p* value	1 year	2 years	*p* value	1 year	2 years	*p* value
All eyes (*n* = 123)	29 (23.6%)	36 (29.3%)	0.21	20 (16.3%)	25 (20.3%)	0.18	23 (18.7%)	20 (16.3%)	0.81	10 (8.1%)	12 (9.7%)	0.51
Baseline VA <70 letters (*n* = 77)	28 (36.4%)	31 (40.3%)	0.77	20 (25.9%)	25 (32.5%)	0.18	14 (18.2%)	10 (12.9%)	0.51	6 (7.8%)	7 (9.1%)	0.62
Baseline VA ≥70 letters (*n* = 46)	1 (2.2%)	5 (10.9%)	0.12	0	0		9 (19.5%)	10 (21.7%)	1.0	4 (8.7%)	5 (10.9%)	1.0

Legend: VA = visual acuity.

**Table 3:** The CRT at baseline, 1 year, and 2 years.

CRT, mean ± SD (median; range) (*μ*m)						
	Baseline	1 year	Change from baseline at 1 year	2 years	Change from baseline at 2 years	*p* value
All eyes (*n* = 123)	440.7 ± 132.5 (430; 114–1000)	368.4 ± 138.21 (350; 50–1500)	−71.8 ± 159.9 (−64.5; −910–720)	350.4 ± 108.3 (332; 178–800)	−90.4 ± 131.1 (−85; −210–476)	<0.0001
Eyes with baseline VA <70 letters (*n* = 77)	464.2 ± 143.9 (460; 114–1000)	384.8 ± 164.6 (350; 50–1500)	−73.2 ± 185.9 (−62; −910–720)	363.5 ± 110.9 (340; 178–800)	−101 ± 136.8 (−90; −172–440	<0.0001
Eyes with baseline VA ≥70 letters (*n* = 46)	401.5 ± 100.5 (410; 207–665)	333 ± 82.8 (330; 186–700)	−68.2 ± 106.1 (−66.5; −97–479)	328.5 ± 100.9 (320; 186–770)	−72.4 ± 119.9 (−58; −210–476)	<0.0001

Legend: VA = visual acuity, CRT = central subfield retinal thickness.

## Data Availability

Data used for the analysis are available from the corresponding author upon reasonable request.
